# Moving Toward Age-Diverse Universities: Perspectives of Admission and Career Service Professionals

**DOI:** 10.1093/geroni/igaa057.1805

**Published:** 2020-12-16

**Authors:** Nancy Morrow-Howell, Natalie Galucia, Emma Swinford, Tanner Meyer

**Affiliations:** 1 Washington University in St. Louis, St. Louis, Missouri, United States; 2 Washington University, St. Louis, Missouri, United States

## Abstract

As age diversity in universities increases in response to demographic shifts, changes in educational practices, programs and policies are needed. To inform these transformations, this research focuses on opportunities and challenges of increasing age-diversity. We conducted 5 focus groups and included 31 professional staff at Washington University in St Louis who work in admissions and career services. A thematic content analysis revealed themes in two main categories: challenges of serving non-traditionally aged students (fitting in, career concerns, acclimating to learning environment and technology, ROI, and ageism) and benefits of older students (intentional students, experienced students, and classroom diversity). Recommendations emerged, including affinity groups, social opportunities which include families, community engagement for job placement, financial aid, targeted outreach to older students, academic flexibility, and administrative support. Findings will be used to advocate for increasing age-diversity among students at Washington University and other institutions in the Age-Friendly Global Network..

